# Increasing incidence and antimicrobial resistance in *Escherichia coli* bloodstream infections: a multinational population-based cohort study

**DOI:** 10.1186/s13756-021-00999-4

**Published:** 2021-09-06

**Authors:** Melissa C. MacKinnon, Scott A. McEwen, David L. Pearl, Outi Lyytikäinen, Gunnar Jacobsson, Peter Collignon, Daniel B. Gregson, Louis Valiquette, Kevin B. Laupland

**Affiliations:** 1 Department of Population Medicine, University of Guelph, 50 Stone Rd E, Guelph, ON N1G 2W1 Canada; 2 Department of Health Security, National Institute for Health and Welfare, Helsinki, Finland; 3 Department of Infectious Diseases, Skaraborg Hospital, Skövde, Sweden; 4 CARe - Center for Antibiotic Resistance Research, Institute of Biomedicine, University of Gothenburg, Gothenburg, Sweden; 5 Department of Infectious Disease and Microbiology, The Canberra Hospital, Garran, ACT Australia; 6 Medical School, Australian National University, Acton, ACT Australia; 7 Departments of Medicine, and Pathology and Laboratory Medicine, University of Calgary, Calgary, AB Canada; 8 Alberta Health Services, Calgary Zone, Calgary, AB Canada; 9 Department of Microbiology-Infectious Diseases, Université de Sherbrooke, Sherbrooke, QC Canada; 10 Department of Medicine, Royal Inland Hospital, Kamloops, BC Canada; 11 Department of Intensive Care Medicine, Royal Brisbane and Women’s Hospital, Brisbane, QLD Australia; 12 Faculty of Health, Queensland University of Technology (QUT), Brisbane, QLD Australia

**Keywords:** Population-based, Bloodstream infection, Bacteremia, *Escherichia coli*, Incidence rate, Antimicrobial resistance, Third-generation cephalosporins

## Abstract

**Background:**

*Escherichia coli* is an important pathogen in humans and is the most common cause of bacterial bloodstream infections (BSIs). The objectives of our study were to determine factors associated with *E. coli* BSI incidence rate and third-generation cephalosporin resistance in a multinational population-based cohort.

**Methods:**

We included all incident *E. coli* BSIs (2014–2018) from national (Finland) and regional (Australia [Canberra], Sweden [Skaraborg], and Canada [Calgary, Sherbrooke, and western interior]) surveillance. Incidence rates were directly age and sex standardized to the European Union 28-country 2018 population. Multivariable negative binomial and logistic regression models estimated factors significantly associated with *E. coli* BSI incidence rate and third-generation cephalosporin resistance, respectively. The explanatory variables considered for inclusion in both models were year (2014–2018), region (six areas), age (< 70-years-old and ≥ 70-years-old), and sex (female and male).

**Results:**

We identified 31,889 *E. coli* BSIs from 40.7 million person-years of surveillance. Overall and third-generation cephalosporin-resistant standardized rates were 87.1 and 6.6 cases/100,000 person-years, respectively, and increased 14.0% and 40.1% over the five-year study. Overall, 7.8% (2483/31889) of *E. coli* BSIs were third-generation cephalosporin-resistant. Calgary, Canberra, Sherbrooke, and western interior had significantly lower *E. coli* BSI rates compared to Finland. The significant association between age and *E. coli* BSI rate varied with sex. Calgary, Canberra, and western interior had significantly greater odds of third-generation cephalosporin-resistant *E. coli* BSIs compared to Finland. Compared to 2014, the odds of third-generation cephalosporin-resistant *E. coli* BSIs were significantly increased in 2016, 2017, and 2018. The significant association between age and the odds of having a third-generation cephalosporin-resistant *E. coli* BSI varied with sex.

**Conclusions:**

Increases in overall and third-generation cephalosporin-resistant standardized *E. coli* BSI rates were clinically important. Overall, *E. coli* BSI incidence rates were 40–104% greater than previous investigations from the same study areas. Region, sex, and age are important variables when analyzing *E. coli* BSI rates and third-generation cephalosporin resistance in *E. coli* BSIs. Considering *E. coli* is the most common cause of BSIs, this increasing burden and evolving third-generation cephalosporin resistance will have an important impact on human health, especially in aging populations.

**Supplementary Information:**

The online version contains supplementary material available at 10.1186/s13756-021-00999-4.

## Background

*Escherichia coli* is the most common cause of bloodstream infections (BSIs) (1–3). Regional population-based studies conducted in Canberra (Australia; 2000–2004), Calgary (Canada; 2000–2006), Funen County (Denmark; 2000–2008), Auckland (New Zealand; 2005–2011), and Skaraborg County (Sweden; 2011–2012, only community-onset *E. coli* BSIs) described annual *E. coli* BSI rates of 28.0–70.2/100,000 population (2, 4–7). A national population-based study from England revealed annual rates of 60.4/100,000 population (04/2012–03/2013) and 63.5/100,000 population (04/2013–03/2014) (8). Recently, some areas worldwide reported an increase in *E. coli* BSI incidence (8–10). The emergence of third-generation cephalosporin-resistant (3GC-R) *E. coli* has also had a major effect on the epidemiology and treatment of these infections worldwide (11). Third-generation cephalosporin-resistant *E. coli* infections are often multidrug-resistant, and in contrast to many other multidrug-resistant bacteria, they mostly emerge in community settings (5, 6, 8, 12).

There is an increasingly large body of literature on *E. coli* BSIs. However, most studies are hospital-based and typically recruit patients from highly selected populations at large tertiary-care centres. In order to determine the incidence rate of *E. coli* BSIs and understand the associated burden of disease, a population-based study is required (13). By using this approach, the population at risk can be defined, and selection bias is minimized by including all *E. coli* BSIs from the population (13). To our knowledge, a multinational population-based study evaluating incidence rates and antimicrobial resistance (AMR) in *E. coli* BSIs has not been previously published.

Using multinational population-based data, we aimed to: 1) evaluate the incidence rate of *E. coli* BSIs and associated factors; and 2) evaluate factors associated with having a 3GC-R *E. coli* BSI.

## Methods

### Study protocol

For this population-based cohort study, we enrolled six participating surveillance areas from the International Bacteremia Surveillance Collaborative based on their willingness to voluntarily participate in the study, and their ability to provide the required data and meet project timelines (14). Finland contributed active national surveillance data. Regional surveillance data were available from areas within Canada (three areas), Australia (one area), and Sweden (one area). From 01/01/2014 to 31/12/2018, all incident episodes of *E. coli* BSIs from residents within the surveillance areas were included, whether they occurred in a hospital or community setting. An incident BSI was defined as growth of *E. coli* from at least one blood culture, and only the first *E. coli* isolate per patient per running year was included (i.e., at least one year of time elapsed between *E. coli* BSIs). The surveillance databases from each area identify an estimated 99% or greater of all positive blood cultures in their residents, except for Canberra Region, where at least 95% are detected (as a private laboratory processes some blood cultures) (14, 15). We retrieved the following data from electronic medical records: year of culture; patient’s sex and age category (< 1-year then deciles until ≥ 90-years); location of onset (hospital-onset or community-onset); and susceptibility to 3GC, ciprofloxacin, gentamicin, trimethoprim/sulfamethoxazole (TMS), and meropenem. Resistance to 3GC was defined according to each area’s established methodology and details are available in Additional file 1. Each area performed susceptibility testing according to their own established protocols. Data for location of onset were not available from Canberra. Susceptibility data for ciprofloxacin, gentamicin, TMS, and meropenem were not available from Canberra and Finland. If the first positive blood culture was obtained at least 48 h after hospital admission or within 48 h of hospital discharge, the BSI was characterized as hospital-onset; otherwise, it was characterized as community-onset (16). Individual surveillance areas compiled data using standardized data summary templates. The following research ethics boards approved the study and granted waivers of informed consent: the Interior Health Research Ethics Board (2013-14-052-I); the University of Guelph Research Ethics Board (2018-10-050); the Conjoint Health Research Ethics Board of the University of Calgary (REB19-1025); the Regional Ethics Board Gothenburg (539–11); the Ethics Committee of the Finnish Institute for Health and Welfare (THL/1349/6.02.00/2019); the ACT (Australian Capital Territory) Health Human Research Ethics Committee (2020.LRE.00115); and Comité d’éthique de la recherche du CIUSSS de l’Estrie—CHUS (Centre intégré universitaire de santé et de services sociaux de l'Estrie—Centre hospitalier universitaire de Sherbrooke) (2011-286, 10-181). Analyses related to mortality in *E. coli* BSIs in this study are documented in a separate manuscript.

### Surveillance populations

The study surveillance populations (2018) included: Calgary Health Region, Canada (1.7 million); Canberra Region, Australia (421,000); country of Finland (5.5 million); Sherbrooke Region, Canada, (166,000); Skaraborg County Health Region, Sweden (267,000); and western interior area of British Columbia, Canada (191,000). Detailed descriptions of each area’s population and surveillance methodology have been previously published (14, 15, 17). Of note, since the time of previous publications, Skaraborg now has two hospitals instead of four.

### Data management and statistical analysis

The data analyses were performed in Stata 15.1 (18). We calculated prevalence for dichotomous variables (sex, location of onset, and AMR data) and to summarize age, we determined the age category that contained the median of the *E. coli* BSI age distribution. Using univariable logistic regression, an odds ratio (OR) was estimated to compare the odds of a BSI being hospital-onset in 3GC-R and 3GC-susceptible (3GC-S) *E. coli* BSIs. We calculated incidence rates by dividing the number of incident *E. coli* BSIs by the surveillance population, which was obtained from individual area census data. To facilitate comparison of incidence rates between different regions and different years, we directly age and sex standardized the incidence rates to the European Union 28-country (EU28) 2018 population (18, 19). Incidence rates for 3GC-R and 3GC-S *E. coli* BSIs were calculated and directly standardized as above. We used a negative binomial regression model to determine factors significantly associated with *E. coli* BSI incidence rates (18, 20). To explore factors significantly associated with having 3GC-R *E. coli* BSIs, we used a logistic regression model (18, 20). For inclusion in each regression model, we considered the following four categorical explanatory variables: year (2014 through 2018); region (six study areas); age (< 70-years-old and ≥ 70-years-old); and sex (female and male). Age was dichotomized using a 70-year-old cut-off based on the structure of our data (collected in 10-year age brackets for adults) and to facilitate modelling the risk factor of elderly age. We performed univariable analysis and assessed for high correlation between explanatory variables using a Phi coefficient (ρ ≥|0.8| represented significant correlation) prior to all explanatory variables being placed in the multivariable regression models. We considered interaction effects between year and region, and age and sex for inclusion in the multivariable regression models due to their epidemiologic and / or biologic plausibility. To remain in the final multivariable model, a variable had to be statistically significant (α = 0.05), part of a significant interaction term, or an important confounder (based on meeting causal criteria and > 20% change in another variable’s coefficient) (20). The final multivariable negative binomial regression model was assessed for goodness-of-fit (deviance goodness-of-fit test and normality of Anscombe residuals), and residuals (Pearson and deviance), leverage and an influence statistic (Cook’s distance) were assessed (20). The final multivariable logistic regression model was assessed for goodness-of-fit (Pearson goodness-of-fit test), and standardized Pearson residuals, leverage and influence statistics (delta-beta, delta-chi^2^, and delta-deviance) were assessed (20). We performed contrasts to explore interaction terms included in the final multivariable models. Incidence rate ratios (IRR) and OR were reported with 95% confidence intervals (CI).

### Missing data

We used a casewise deletion method to manage missing data, where incident *E. coli* BSIs were removed from specific analyses if data were incomplete. A total of 17 *E. coli* BSIs were removed from the main descriptive and regression modelling analyses due to missing data for age, sex, and third-generation cephalosporin susceptibility. Location of onset was missing for 983 incident *E. coli* BSIs. Data regarding susceptibility to ciprofloxacin, gentamicin, TMS, and meropenem were missing for 25,622, 25,614, 25,606 and 25,739 incident *E. coli* BSIs, respectively.

## Results

**Fig. 1:**
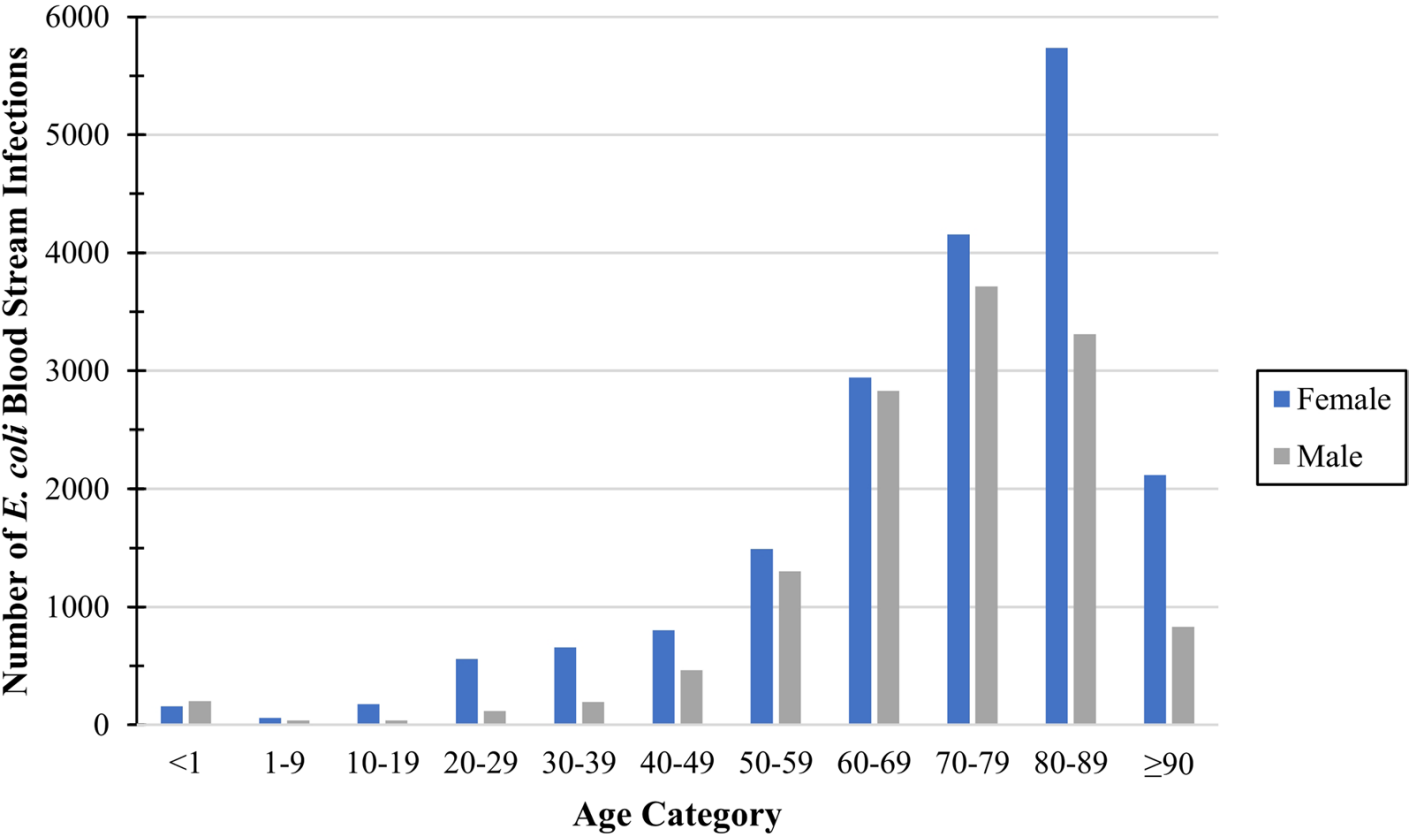
Number of *E. coli* bloodstream infections by age category and sex

We identified 31,889 incident *E. coli* BSIs from 40.7 million patient-years of follow-up during the five-year study; 59.1% (18,856/31,889) of those were in females. The median age range for *E. coli* BSI patients was 70–79-years-old, and the distribution was left-skewed (Fig. 1). Overall, 7.8% (2483/31889) of *E. coli* BSIs were 3GC-R ranging from 6.1% (1510/24296) in Finland to 17.8% (671/3773) in Calgary. Most *E. coli* BSIs were community-onset (82.2%; 25,417/30,923), and this was lowest in Finland (80.8%, 19,909/24,629) and highest in Skaraborg (92.1%, 1241/1347). A 3GC-R *E. coli* BSI was at greater odds of being hospital-onset compared to 3GC-S (OR:1.68, 95%CI:1.53–1.85, *p* < 0.001). The proportion of *E. coli* BSIs that were resistant to ciprofloxacin, gentamicin, TMS, and meropenem was 23.4% (1473/6284), 11.2% (702/6292), 26.1% (1642/6300), and 0.1% (6/6167), respectively. Additional file 2 contains regional data for AMR and location of onset.

**Fig. 2:**
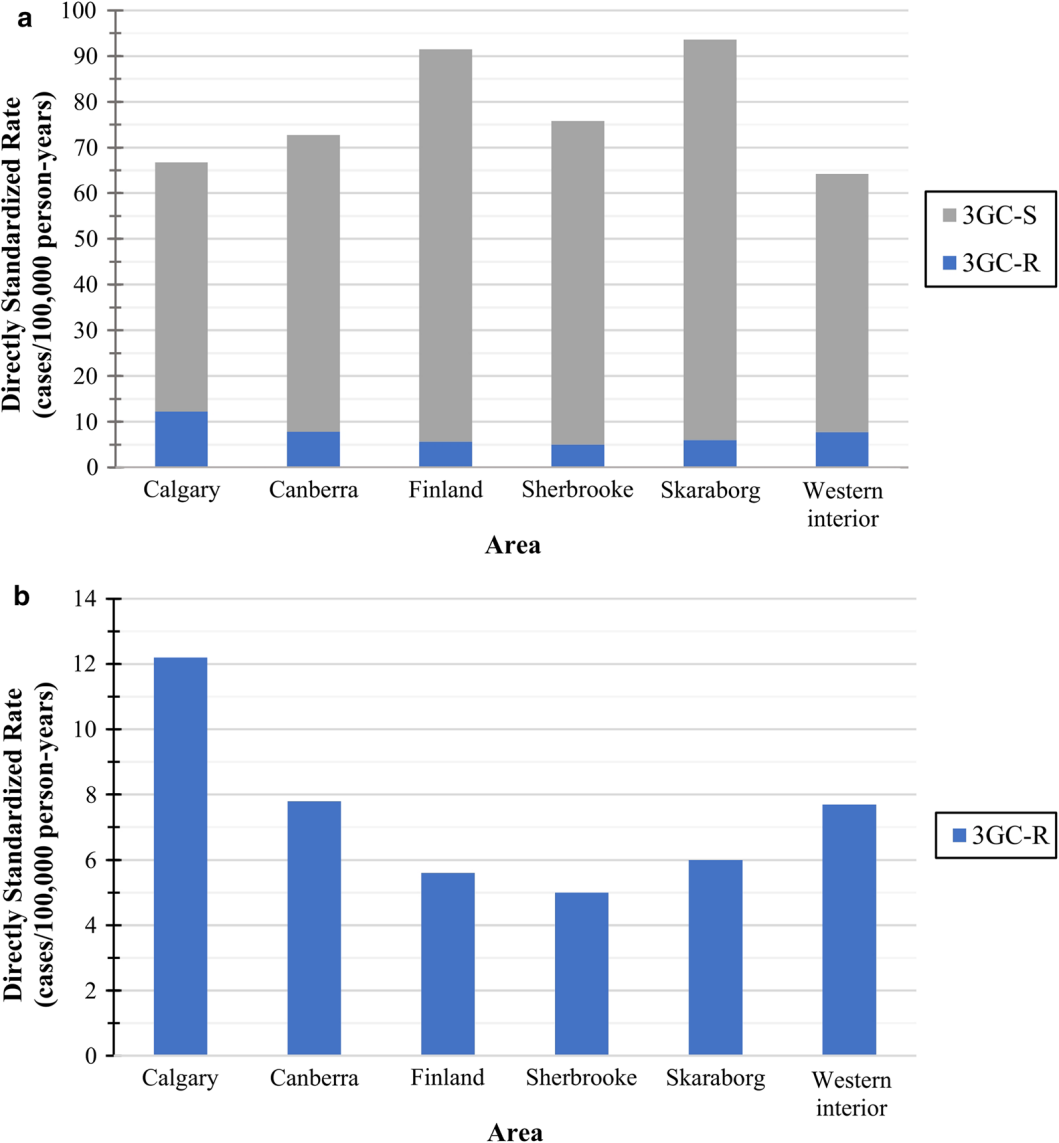
Directly age and sex standardized *E. coli* bloodstream infection incidence rates by area^a,b^. **a** Overall, and third-generation cephalosporin-resistant and -susceptible standardized incidence rates by area^a,b^. **b** Third-generation cephalosporin-resistant standardized incidence rates by area^a,b^. *3GC-R* Third-generation cephalosporin-resistant; *3GC-S* Third-generation cephalosporin-susceptible. ^a^Standard population—European Union 28-country 2018 population. ^b^Standardized *E. coli* bloodstream infection rates by area are available in Additional file 4

**Fig. 3:**
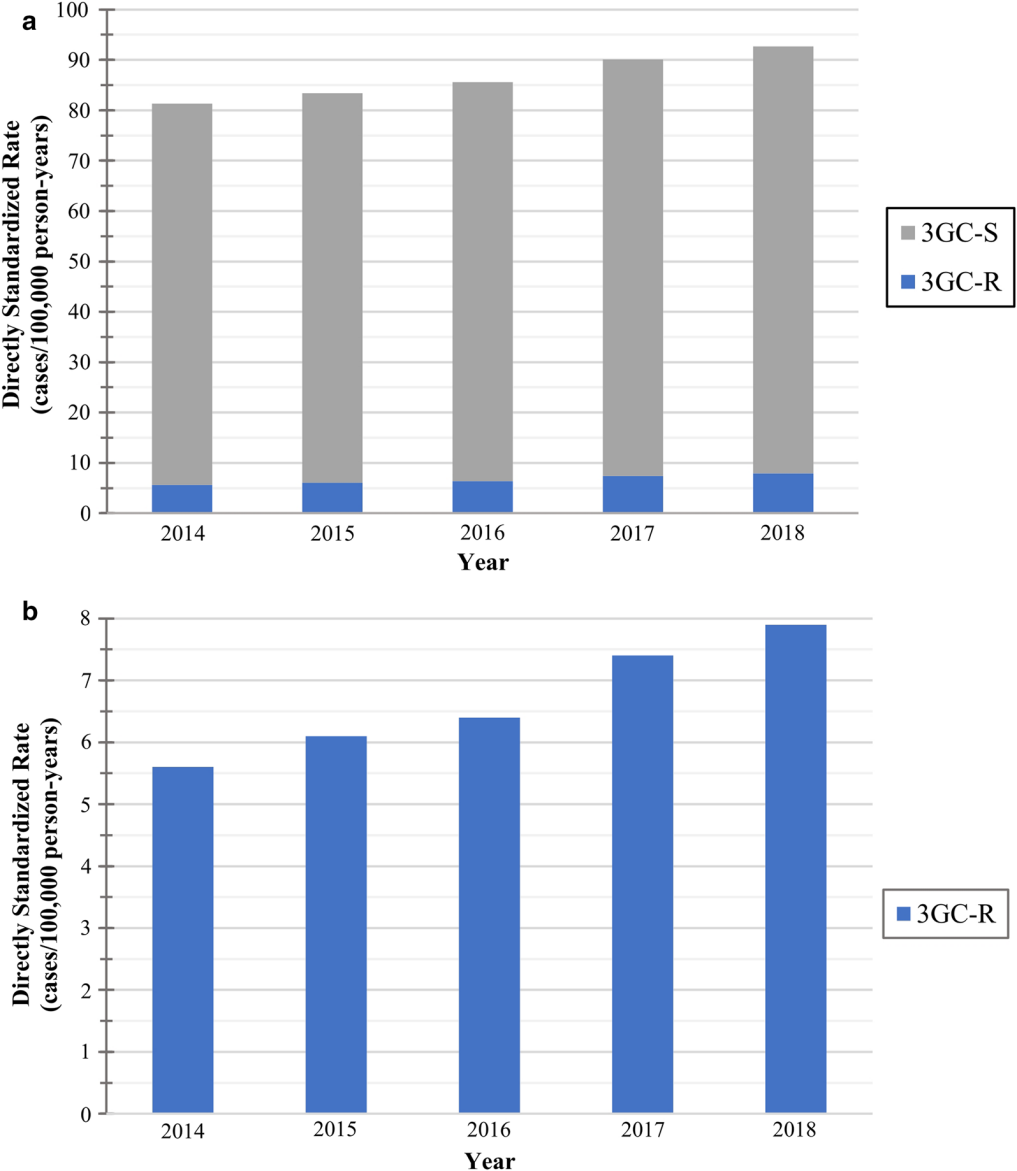
Directly age and sex standardized *E. coli* bloodstream infection incidence rates by year^a,b^. **a** Overall, and third-generation cephalosporin-resistant and -susceptible standardized incidence rates by year^a,b^. **b** Third-generation cephalosporin-resistant standardized incidence rates by year^a,b^. *3GC-R* Third-generation cephalosporin-resistant; *3GC-S* Third-generation cephalosporin-susceptible. ^a^Standard population—European Union 28-country 2018 population. ^b^Standardized *E. coli* bloodstream infection rates by year are available in Additional file 4

The overall crude *E. coli* BSI rate was 78.4 cases/100,000 person-years (see Additional file 3). The overall annual directly age and sex standardized rate was 87.1 *E. coli* BSI/100,000 population, which was lowest in western interior and highest in Skaraborg (64.2 and 93.6 cases/100,000 population) (Fig. 2a). For 3GC-R *E. coli* BSIs, the overall annual standardized rate was 6.6 cases/100,000 population ranging from 5.0 cases/100,000 population in Sherbrooke to 12.2 cases/100,000 population in Calgary (Fig. 2a, b). From 2014 to 2018, the overall and 3GC-R annual standardized rates increased by 14.0% and 41.1%, respectively (Fig. 3a, b). Additional file 4 contains regional and annual standardized overall, 3GC-R, and 3GC-S *E. coli* BSI rates.

With univariable negative binomial regression analysis, there was a significant association between age and *E. coli* BSI rate, but no variation by year, region, or sex (see Additional file 5). Our multivariable negative binomial model for *E. coli* BSI rates included region, and an interaction between age and sex (Table 1). Calgary, Canberra, Sherbrooke, and western interior had significantly lower rates of *E. coli* BSI compared to Finland (Table 1). In terms of the interaction between sex and age, males or females that were ≥ 70-years-old had significantly higher *E. coli* BSI rates compared to < 70-year-old males or females (Table 2). However, while females < 70-years-old had significantly higher *E. coli* BSI rates compared to males < 70-years-old, there was no significant difference in rates between females and males that were ≥ 70-years-old (Table 2).

**Table 1:** Multivariable negative binomial regression model results estimating associations between explanatory variables and *E. coli* BSI rates^a,b^

Variable	aIRR	95% CI	*p*-value
Region			< 0.001
Finland	1.00	Referent	
Calgary	0.71	0.65–0.77	< 0.001
Canberra	0.71	0.65–0.79	< 0.001
Sherbrooke	0.86	0.77–0.96	< 0.001
Skaraborg	1.05	0.96–1.15	0.312
Western interior	0.73	0.65–0.82	< 0.001
Sex			
Female	1.00	Referent	
Male	0.74^c^	0.67–0.80	< 0.001
Age category			
< 70-years-old	1.00	Referent	
≥ 70-years-old	9.37^c^	8.63–10.19	< 0.001
Interaction—sex and age			
Male and ≥ 70	1.39^c^	1.23–1.57	< 0.001

*BSI* Bloodstream infection; *aIRR* Adjusted incidence rate ratio; *CI* Confidence interval

^a^Overdispersion parameter 0.014, 95% CI:0.008–0.025, *p* < 0.001

^b^Model fit the data based on normally distributed Anscombe residuals and non-significant deviance goodness-of-fit test (*p* = 0.24)

^c^Exponentiated coefficients are not true aOR due to interaction term—see contrasts in Table 2

**Table 2:** Results for contrasts examining interactions between sex and age based on multivariable negative binomial regression model^a^

Contrast statement	IRR	95% CI	*p*-value
Females ≥ 70 compared to females < 70	9.37	8.63–10.19	< 0.001
Males ≥ 70 compared to females < 70	9.61	8.83–10.45	< 0.001
Females ≥ 70 compared to males < 70	12.72	11.68–13.86	< 0.001
Males ≥ 70 compared to males < 70	13.04	11.96–14.22	< 0.001
Females ≥ 70 compared to males ≥ 70	0.98	0.90–1.06	0.558
Females < 70 compared to males < 70	1.36	1.24–1.48	< 0.001

*IRR* Incidence rate ratio; *CI* Confidence interval

^a^Multivariable negative binomial regression model estimating the associations between the explanatory variables (region, sex, and age) and *E. coli* bloodstream infection incidence rate (Table 1)

Region, year, age, and sex were significantly associated with having 3GC-R *E. coli* BSIs using univariable logistic regression analysis (see Additional file 6). Our multivariable logistic model for having 3GC-R *E. coli* BSIs included region, year, and an interaction between age and sex (Table 3). Calgary, Canberra, and western interior had significantly greater odds of 3GC-R *E. coli* BSIs than Finland (Table 3). Compared to 2014, the odds of 3GC-R *E. coli* BSIs were significantly increased in 2016, 2017, and 2018 (Table 3). In terms of the interaction between sex and age, males in either age category were at significantly increased odds of having 3GC-R *E. coli* BSIs compared to females in either age category (Table 4). However, while males < 70-years-old were at significantly increased odds of having 3GC-R *E. coli* BSIs compared to males ≥ 70-years-old, there was no significant difference between females in different age categories (Table 4).

**Table 3:** Multivariable logistic regression model results estimating associations between explanatory variables and having 3GC-R *E. coli* BSI^a^

Variable	aOR	95% CI	*p*-value
Region			< 0.001
Finland	1.00	Referent	
Calgary	3.25	2.94–3.59	< 0.001
Canberra	1.87	1.52–2.31	< 0.001
Sherbrooke	1.04	0.75–1.45	0.796
Skaraborg	1.02	0.81–1.27	0.875
Western interior	2.08	1.61–2.70	< 0.001
Year			< 0.001
2014	1.00	Referent	
2015	1.07	0.92–1.23	0.388
2016	1.15	1.00–1.33	0.048
2017	1.23	1.07–1.41	0.003
2018	1.31	1.15–1.50	< 0.001
Sex			
Female	1.00	Referent	
Male	1.83^b^	1.61–2.09	< 0.001
Age category			
< 70-years-old	1.00	Referent	
≥ 70-years-old	1.05^b^	0.93–1.19	0.420
Interaction—sex and age			
Male and ≥ 70	0.84^b^	0.71–0.999	0.048

*3GC-R* Third-generation cephalosporin-resistant; *BSI* Bloodstream infection; *aOR* Adjusted odds ratio; *CI* Confidence interval

^a^Model fit the data based on non-significant Pearson goodness-of-fit test (*p* = 0.072)

^b^Exponentiated coefficients are not true aOR due to interaction term—see contrasts in Table 4

**Table 4:** Results for contrasts examining interactions between sex and age based on multivariable logistic regression model^a^

Contrast statement	OR	95% CI	*p*-value
Males < 70 compared to females < 70	1.83	1.61–2.09	< 0.001
Males ≥ 70 compared to females < 70	1.62	1.44–1.84	< 0.001
Females ≥ 70 compared to females < 70	1.05	0.93–1.19	0.420
Males < 70 compared to females ≥ 70	1.74	1.55–1.96	< 0.001
Males ≥ 70 compared to females ≥ 70	1.54	1.39–1.72	< 0.001
Males < 70 compared to males ≥ 70	1.13	1.00–1.27	0.045

*OR* Odds ratio; *CI* Confidence interval

^a^Multivariable logistic regression model estimating the associations between the explanatory variables (region, year, sex, and age) and having a third-generation cephalosporin-resistant *E. coli* bloodstream infection (Table 3)

## Discussion

Our study provides several notable contributions to the *E. coli* BSI literature. It is the first multinational population-based study of *E. coli* BSIs, which included all incident cases over a five-year period from six areas in four countries on three continents. The population-based design of the study allowed us to thoroughly capture both community-onset and hospital-onset *E. coli* BSIs; a very important aspect for *E. coli* BSIs since the vast majority are community-onset, including 82.2% in our study. The five-year timeframe of the study and multinational design allowed us to explore annual and regional variation in the incidence rate and 3GC resistance of *E. coli* BSIs. We incorporated the demographic factors of age and sex using several techniques, including: direct age and sex standardization of *E. coli* BSI rates to facilitate comparisons of rates between different regions and different years, and with future studies; and inclusion of age and sex in our regression models.

In population-based studies, the incidence rate of infection provides insight into the burden of the disease being studied (13). The overall crude and directly age and sex standardized rates of *E. coli* BSIs in our study were 78.4 and 87.1 cases/100,000 person-years, respectively, and included 31,889 incident *E. coli* BSIs from 2014 to 2018. We found a higher standardized BSI rate than rates from all previously published population-based studies (Table 5). Our crude BSI rate was also higher than all crude rates (Table 5). We need to note, however, that these rate comparisons are general in nature and should be interpreted cautiously. These rates are from different time periods, and there are differences in demographics between the populations that are unaccounted for when crude rates or rates standardized with a different standard population are compared. Previous population-based studies reported prevalences of 3GC-R *E. coli* BSIs that varied from 1–10.4% (5, 6, 8, 21), which is similar to our overall prevalence of 7.8%.

**Table 5:** Study details and *E. coli* bloodstream infection incidence rates from previously published population-based studies

Study location	Study dates	Number of *E. coli* BSI	Incidence rate (cases/100,000 person-years)	Type of incidence rate
Olmsted County, USA (21)	1998–2007	461	41.4	Standardized^a^
Canberra, Australia (4)	2000–2004	515	28	Crude
Funen County, Denmark (2)	2000–2002	811	70.2	Crude
	2003–2005	718	61.8	Crude
	2006–2008	671	56.7	Crude
mid-Norway (9)	2002–2013	686	80	Crude
	2002–2013	686	74	Standardized^b^
Calgary, Canada (5)	2000–2006	2,368	30.3	Crude
Finland (national) (1)	2004–2007	9,190	44	Crude
Auckland, New Zealand (6)	2006–2011	1,507	52	Crude
Skaraborg County, Sweden (7)	2011–2012	104^c^	67.0	Crude
Two rural Thai provinces (10)	2008	373	32.9	Crude
	2014	593	51.6	Crude
England (national) (8)	04/2012–03/2013	32,309	60.4	Crude
	04/2013–03/2014	34,203	63.5	Crude

*BSI* Bloodstream infection

^a^USA 2000 white population standard

^b^Norway 2010 population

^c^Only community-onset *E. coli* BSI with severe sepsis

Both the *E. coli* BSI rate and 3GC resistance had significant regional differences. The Scandinavian areas had higher *E. coli* BSI rates but lower odds of 3GC-R *E. coli* BSIs. The opposite was seen in Canberra, Calgary and western interior. Region likely serves as a proxy variable for many unmeasured regional and population characteristics. Therefore, our study is not able to determine the underlying reason(s) for these regional differences, which also could be different on a national or regional scale. In theory, some regional differences could be due to varying degrees of capturing positive *E. coli* blood cultures by different areas (surveillance system coverage), but this is unlikely with our enrolled surveillance areas because they all captured at least 95% of all positive blood cultures in their residents. To explore the underlying reason(s) for the regional differences, future studies could evaluate culturing rate, surveillance system coverage, healthcare practices (including antimicrobial use), ethnicity, socioeconomic status, and climate, among other factors.

Over the 5-year study period, we identified a clinically important 14.0% increase in the overall standardized *E. coli* BSI rate but did not find significant annual variation in overall *E. coli* BSI rate with negative binomial regression analysis. Previous investigations from areas enrolled in the current study reported overall crude rates of 28, 30.3, 44, 67 cases/100,000 person-years for Canberra (2000–2004), Calgary (2000–2006), Finland (2004–2007), Skaraborg (2011–2012, community-onset only), respectively (1, 4, 5, 7). The rates reported in our study represent notable rate increases between each of the study pairs of 71%, 52%, 104%, 40% in Canberra, Calgary, Finland, and Skaraborg, respectively. It appears that the rate of increase was likely even higher prior to the 2014 beginning of our study. We found a considerable (41.1%) increase in the 3GC-R *E. coli* BSI rate over our 5-year study period and an increase in the odds of having 3GC-R *E. coli* BSIs in 2016–2018 compared to 2014. The association between year and having 3GC-R *E. coli* BSIs did not depend on the region being considered, since the interaction term between year and region was not statistically significant. This increase in the proportion of 3GC-R *E. coli* BSIs will have important impacts on patient burden, healthcare burden and empirical antimicrobial therapy guidelines since 3GC-R and extended-spectrum beta-lactamase-producing *E. coli* BSIs have been associated with increased treatment failure, mortality, length of hospital stay, and hospital costs (22–25).

Age and sex are demographic factors that are known to impact the rate and odds of disease and may do so through interaction, such that the relationship depends on which combination of sex and age are being considered (8, 15). Our multivariable negative binomial regression model for *E. coli* BSI rate and multivariable logistic regression model for having 3GC-R *E. coli* BSIs both included a significant interaction term between age and sex. We did not identify a previous population-based study that explored interaction between age and sex, however, previous studies have shown that females and older patients had an increased rate of *E. coli* BSIs (1, 3, 5, 9, 26). This reinforces the importance of considering interaction terms between age and sex for inclusion in multivariable models.

There are some limitations to this study. We did not have information to further categorize the community-onset BSIs into community-acquired and healthcare-acquired. Culturing rates were not available for the enrolled areas (27). We did not have access to information regarding comorbidities, source of *E. coli* BSI, or treatment. The laboratory methodology was based on each area’s protocol, and therefore, there may have been some small methodological differences. Population data were provided by each area and although they all have highly developed procedures for census and population estimation, there are likely some methodological differences between areas. All of the areas were from high-income countries limiting the generalizability of our results to comparable countries (28). There is one *E. coli* BSI population-based study from two rural provinces in Thailand, an upper-middle-income country (10); however, more population-based research on *E. coli* BSIs in low, lower-middle, and upper-middle-income countries is needed to understand the global burden of disease (28).

## Conclusions

Our multinational population-based study demonstrated the substantial and increasing burden of *E. coli* BSIs. During the 5-year study, there was a clinically important increase in the overall and 3GC-R standardized *E. coli* BSI rates and a noteworthy significant increase in the odds of having 3GC-R *E. coli* BSIs in 2016–2018. There were significant regional, sex, and age differences in both the rate and 3GC resistance of *E. coli* BSIs. These findings highlight the difficulty in comparing and extrapolating results from single-centre studies where there are likely differences in unmeasured regional factors and demographics. Considering *E. coli* is the most common cause of BSIs, this increasing burden and evolving 3GC resistance will have an important impact on human health, especially in aging populations.

## Supplementary Information

**Additional file 1**. Table summarizing the methodology used to determine susceptibility to third-generation cephalosporins by the enrolled areas.

**Additional file 2**. Table containing proportion of E. coli bloodstream infections by region that were resistant to third-generation cephalosporins, ciprofloxacin, gentamicin, trimethoprim/sulfamethoxazole, or meropenem and location of onset.

**Additional file 3**. Table containing counts of incident E. coli bloodstream infections, length of patient follow-up and crude rates of E. coli bloodstream infections.

**Additional file 4**. Table containing directly age and sex standardized E. coli bloodstream infection incidence for overall, third-generation cephalosporin-resistant and susceptible E. coli bloodstream infections

**Additional file 5**. Table containing the crude incidence rate ratios for the univariable negative binomial regression models estimating associations between E. coli bloodstream infection rates, and region, year, sex and age.

**Additional file 6**. Table containing the crude odds ratios for the univariable logistic regression models estimating associations between having a third-generation cephalosporin-resistant E. coli bloodstream infection, and region, year, sex and age.

## Data Availability

The aggregated datasets analyzed during the current study may be available from the corresponding author on reasonable request.

## Abbreviations

3GC: Third-generation cephalosporin
3GC-R: Third-generation cephalosporin-resistant
3GC-S: Third-generation cephalosporin-susceptible
AMR: Antimicrobial resistance
BSI: Bloodstream infection
BSIs: Bloodstream infections
*E. coli*: *Escherichia coli*
EU28: European Union 28-country
IRR: Incidence rate ratio
OR: Odds ratio
TMS: Trimethoprim/sulfamethoxazole
