# Supplementary material for: Increasing incidence and antimicrobial resistance in Escherichia coli bloodstream infections: a multinational population-based cohort study
Source: Antimicrob Resist Infect Control. 2021 Sep 6;10:131. doi: 10.1186/s13756-021-00999-4 (PMC8422618; doi:10.1186/s13756-021-00999-4)
Supplement: Supplementary file 1 — Additional file 1. Table summarizing the methodology used to determine susceptibility to third-generation cephalosporins by the enrolled areas. [file 13756_2021_999_MOESM1_ESM.pdf]

Additional file 1 – Table summarizing the methodology used to determine susceptibility to third-generation cephalosporins by the areas enrolled in a multinational population-based cohort study of *E. coli* bloodstream infections (2014 to 2018)

Region	Methodology to determine susceptibility to third-generation cephalosporins[a]		
	**Antimicrobials assessed**	**Type of clinical breakpoint used**	**Antimicrobial susceptibility test**
Calgary	Ceftriaxone and/or ceftazidime	CLSI	Vitek® 2[b] broth microdilution
Canberra	Ceftriaxone and/or ceftazidime	EUCAST	Vitek® 2[b] broth microdilution
Finland	Ceftriaxone and/or ceftazidime and/or cefotaxime	EUCAST	Vitek® 2[b] broth microdilution and disk diffusion
Sherbrooke	Ceftriaxone and/or ceftazidime and/or cefixime	CLSI	Vitek® 2[b] broth microdilution
Skaraborg	Ceftazidime and/or cefotaxime	EUCAST	Disk diffusion
Western interior	Ceftriaxone and/or cefotaxime	CLSI	Vitek® 2[b] broth microdilution

CLSI – Clinical and Laboratory Standards Institute; EUCAST – European Committee on Antimicrobial Susceptibility Testing

[a] Intermediate isolates were considered resistant for data collection
[b] bioMérieux
