# Supplementary material for: Increasing incidence and antimicrobial resistance in Escherichia coli bloodstream infections: a multinational population-based cohort study
Source: Antimicrob Resist Infect Control. 2021 Sep 6;10:131. doi: 10.1186/s13756-021-00999-4 (PMC8422618; doi:10.1186/s13756-021-00999-4)
Supplement: Supplementary file 2 — Additional file 2. Table containing proportion of E. coli bloodstream infections by region that were resistant to third-generation cephalosporins, ciprofloxacin, gentamicin, trimethoprim/sulfamethoxazole, or meropenem and location of onset. [file 13756_2021_999_MOESM2_ESM.pdf]

Additional file 2 – Table containing proportion of *E. coli* bloodstream infections by region that were resistant to third-generation cephalosporins, ciprofloxacin, gentamicin, trimethoprim sulfamethoxazole, or meropenem and location of onset based on data from the multinational population-based cohort study (2014 to 2018)

Region	Proportion resistant to 3GC (#R/Total)	Proportion resistant to ciprofloxacin (#R/Total)	Proportion resistant to gentamicin (#R/Total)	Proportion resistant to TMS (#R/Total)	Proportion resistant to meropenem (#R/Total)	Proportion community-onset (Community-onset/Total)	Proportion hospital-onset (Hospital-onset/Total)
Calgary	17.8% (671/3773)	29.1% (1095/3770)	13.7% (517/3775)	30.8% (1167/3784)	0.1% (4/3780)	85.6% (3228/3773)	14.4% (545/3773)
Canberra	11.1% (107/966)	n/a	n/a	n/a	n/a	n/a	n/a
Finland	6.1% (1510/24629)	n/a	n/a	n/a	n/a	80.8% (19909/24629)	19.2% (4720/24629)
Sherbrooke	6.5% (39/596)	13.1% (78/596)	6.4% (38/594)	16.0% (95/594)	0.2% (1/462)	89.3% (532/596)	10.7% (64/596)
Skaraborg	6.5% (87/1347)	11.4% (154/1347)	7.0% (94/1347)	18.3% (246/1347)	0.07% (1/1347)	92.1% (1241/1347)	7.9% (106/1347)
Western interior	11.9% (69/578)	25.6% (146/571)	9.2% (53/576)	23.3% (134/575)	0.0% (0/578)	87.7% (507/578)	12.3% (71/578)
Total	7.8% (2483/31889)	23.4% (1473/6284)	11.2% (702/6292)	26.1% (1642/6300)	0.1% (6/6167)	82.2% (25417/30923)	17.8% (5506/30923)

3GC – Third-generation cephalosporins; R – Resistant; TMS – Trimethoprim/sulfamethoxazole; n/a – Data not available
