# Supplementary material for: Increasing incidence and antimicrobial resistance in Escherichia coli bloodstream infections: a multinational population-based cohort study
Source: Antimicrob Resist Infect Control. 2021 Sep 6;10:131. doi: 10.1186/s13756-021-00999-4 (PMC8422618; doi:10.1186/s13756-021-00999-4)
Supplement: Supplementary file 3 — Additional file 3. Table containing counts of incident E. coli bloodstream infections, length of patient follow-up and crude rates of E. coli bloodstream infections. [file 13756_2021_999_MOESM3_ESM.pdf]

<u>Additional file 3</u> – Table containing counts of incident *E. coli* bloodstream infections, length of patient follow-up and crude rates of *E. coli* bloodstream infections based on data from a multinational population-based cohort study (2014 to 2018)

**Region**	**Count of incident *E. coli* BSI cases**	**Length of follow-up in patient-years**	**Crude Rate of *E. coli* BSI[1]**
Calgary	3773	8185926	46.1
Canberra	966	2011356	48.0
Finland	24629	27426758	89.8
Sherbrooke	596	820430	72.7
Skaraborg	1347	1319420	102.1
Western interior	578	924714	62.5
Total	31889	40688604	78.4

BSI – Bloodstream infection

[1]Units for the rates are cases/100,000 person-years
