# Supplementary material for: Increasing incidence and antimicrobial resistance in Escherichia coli bloodstream infections: a multinational population-based cohort study
Source: Antimicrob Resist Infect Control. 2021 Sep 6;10:131. doi: 10.1186/s13756-021-00999-4 (PMC8422618; doi:10.1186/s13756-021-00999-4)
Supplement: Supplementary file 4 — Additional file 4. Table containing directly age and sex standardized E. coli bloodstream infection incidence for overall, third-generation cephalosporin-resistant and susceptible E. coli bloodstream infections [file 13756_2021_999_MOESM4_ESM.pdf]

Additional file 4 – Table containing directly age and sex standardized *E. coli* bloodstream infection incidence for overall, third-generation cephalosporin-resistant and susceptible *E. coli* bloodstream infections based on data from a multinational population-based cohort study (2014 to 2018)

**Variable**	**Directly[1] Standardized Rate[2] (Rank[3])**		
	**Overall**	**3GC-R**	**3GC-S**
**Region**			
Calgary	66.7 (5)	12.2 (1)	54.5 (6)
Canberra	72.7 (4)	7.8 (2)	64.9 (4)
Finland	91.6 (2)	5.6 (5)	85.9 (2)
Sherbrooke	75.8 (3)	5.0 (6)	70.8 (3)
Skaraborg	93.6 (1)	6.0 (4)	87.6 (1)
Western interior	64.2 (6)	7.7 (3)	56.5 (5)
**Year**			
2014	81.3	5.6	75.7
2015	83.5	6.1	77.3
2016	85.6	6.4	79.2
2017	90.1	7.4	82.7
2018	92.7	7.9	84.8
**Total**			
Overall	87.1	6.6	80.5

3GC-R – Third-generation cephalosporin-resistant; 3GC-S – Third-generation cephalosporin-susceptible; CI – Confidence interval

[1]Standard population – EU-28 2018 population

[2]Units for the rates are cases/100,000 person-years

[3]Relative ranking of rates for regions from highest (1) to lowest (6)
