# Supplementary material for: Increasing incidence and antimicrobial resistance in Escherichia coli bloodstream infections: a multinational population-based cohort study
Source: Antimicrob Resist Infect Control. 2021 Sep 6;10:131. doi: 10.1186/s13756-021-00999-4 (PMC8422618; doi:10.1186/s13756-021-00999-4)
Supplement: Supplementary file 5 — Additional file 5. Table containing the crude incidence rate ratios for the univariable negative binomial regression models estimating associations between E. coli bloodstream infection rates, and region, year, sex and age. [file 13756_2021_999_MOESM5_ESM.pdf]

Additional file 5 – Table containing the crude incidence rate ratios (95% confidence intervals and p-values) for the univariable negative binomial regression models estimating associations between *E. coli* bloodstream infection rates, and region, year, sex and age based on data from a multinational population-based cohort study (2014 to 2018).

Variable	IRR	95% CI	p-value
**Region**			0.596
Finland	1.00	referent	
Calgary	0.68	0.36 – 1.27	0.225
Canberra	0.83	0.44 – 1.57	0.569
Sherbrooke	0.77	0.41 – 1.46	0.426
Skaraborg	0.99	0.52 – 1.86	0.970
Western interior	0.61	0.32 – 1.15	0.128
**Year**			0.998
2014	1.00	referent	
2015	1.01	0.55 – 1.82	0.978
2016	0.97	0.54 – 1.75	0.923
2017	0.99	0.55 – 1.79	0.979
2018	1.07	0.59 – 1.93	0.823
**Sex**			
Female	1.00	referent	
Male	1.00	0.69 – 1.45	0.990
**Age Category**			
<70-years-old	1.00	referent	
≥70-years-old	10.76	9.74 – 11.89	< 0.001

IRR – Incidence rate ratio; CI – Confidence interval
