# Supplementary material for: Increasing incidence and antimicrobial resistance in Escherichia coli bloodstream infections: a multinational population-based cohort study
Source: Antimicrob Resist Infect Control. 2021 Sep 6;10:131. doi: 10.1186/s13756-021-00999-4 (PMC8422618; doi:10.1186/s13756-021-00999-4)
Supplement: Supplementary file 6 — Additional file 6. Table containing the crude odds ratios for the univariable logistic regression models estimating associations between having a third-generation cephalosporin-resistant E. coli bloodstream infection, and region, year, sex and age. [file 13756_2021_999_MOESM6_ESM.pdf]

<u>Additional file 6</u> – Table containing the crude odds ratios (95% confidence intervals and p-values) for the univariable logistic regression models estimating associations between having a third-generation cephalosporin-resistant *E. coli* bloodstream infection, and region, year, sex and age based on data from a multinational population-based cohort study (2014 to 2018)

**Variable**	**OR**	**95% CI**	**p-value**
**Region**			< 0.0001
Finland	1.00	referent	
Calgary	3.31	3.00 – 3.65	<0.001
Canberra	1.91	1.55 – 2.35	<0.001
Sherbrooke	1.07	0.77 – 1.40	0.678
Skaraborg	1.06	0.85 – 1.32	0.626
Western interior	2.08	1.61 – 2.68	<0.001
**Year**			0.0010
2014	1.00	referent	
2015	1.08	0.94 – 1. 25	0.272
2016	1.12	0. 97 – 1.29	0.109
2017	1.23	1.07 – 1.40	0.003
2018	1.29	1.13 – 1.47	<0.001
**Sex**			
Female	1.00	referent	
Male	1.70	1. 57 – 1.85	<0.0001
**Age Category**			
<70-years-old	1.00	referent	
≥70-years-old	0.83	0.77 – 0.91	< 0.0001

OR – Odds ratio; CI – Confidence interval
